# Supplementary figures and images for: Staging Encystation Progression in Giardia lamblia Using Encystation-Specific Vesicle Morphology and Associating Molecular Markers
Source: Front Cell Dev Biol. 2021 Apr 27;9:662945. doi: 10.3389/fcell.2021.662945 (PMC8111296; doi:10.3389/fcell.2021.662945)

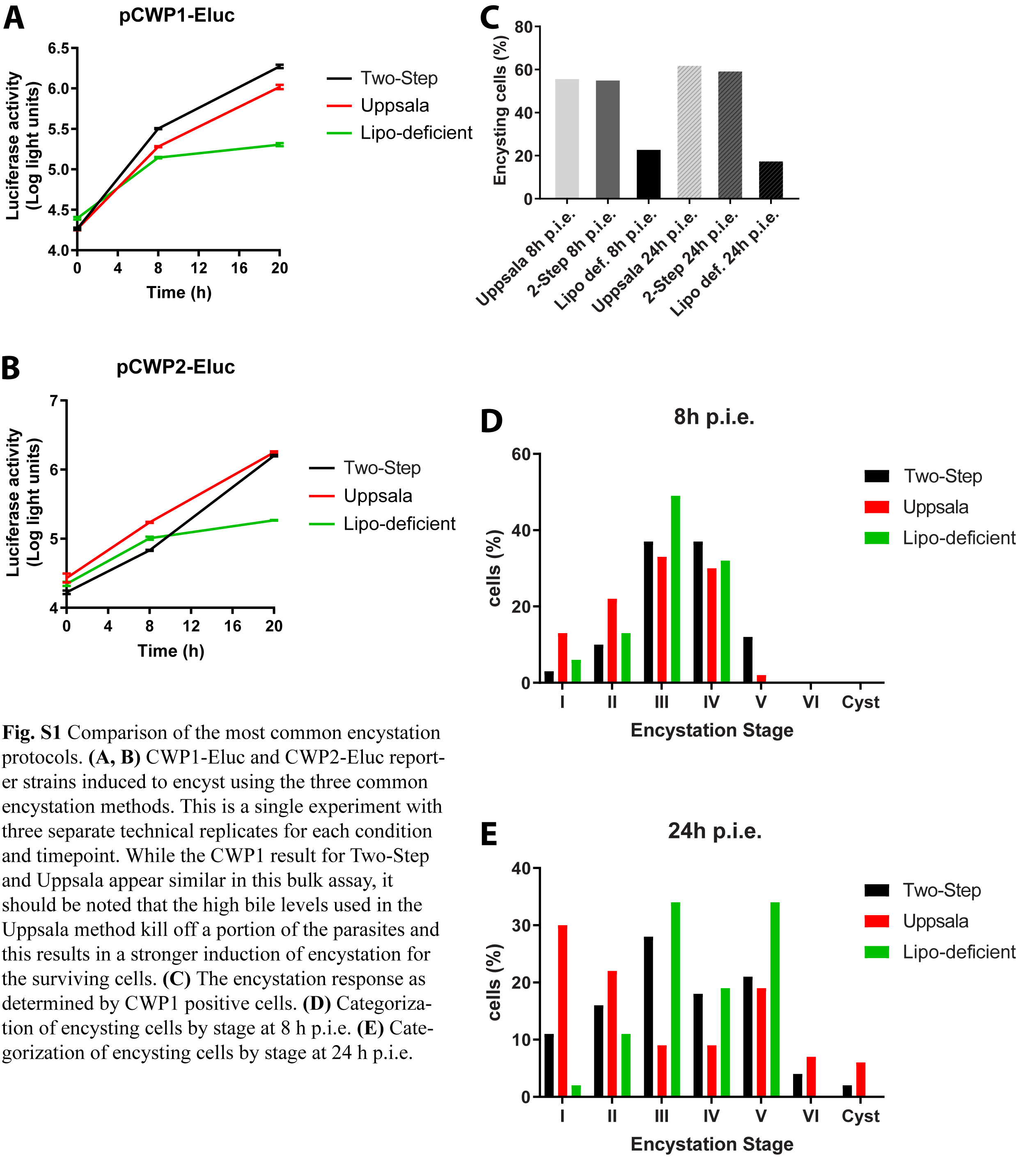

Supplement: Supplementary file 1 [file Image_1.tif]

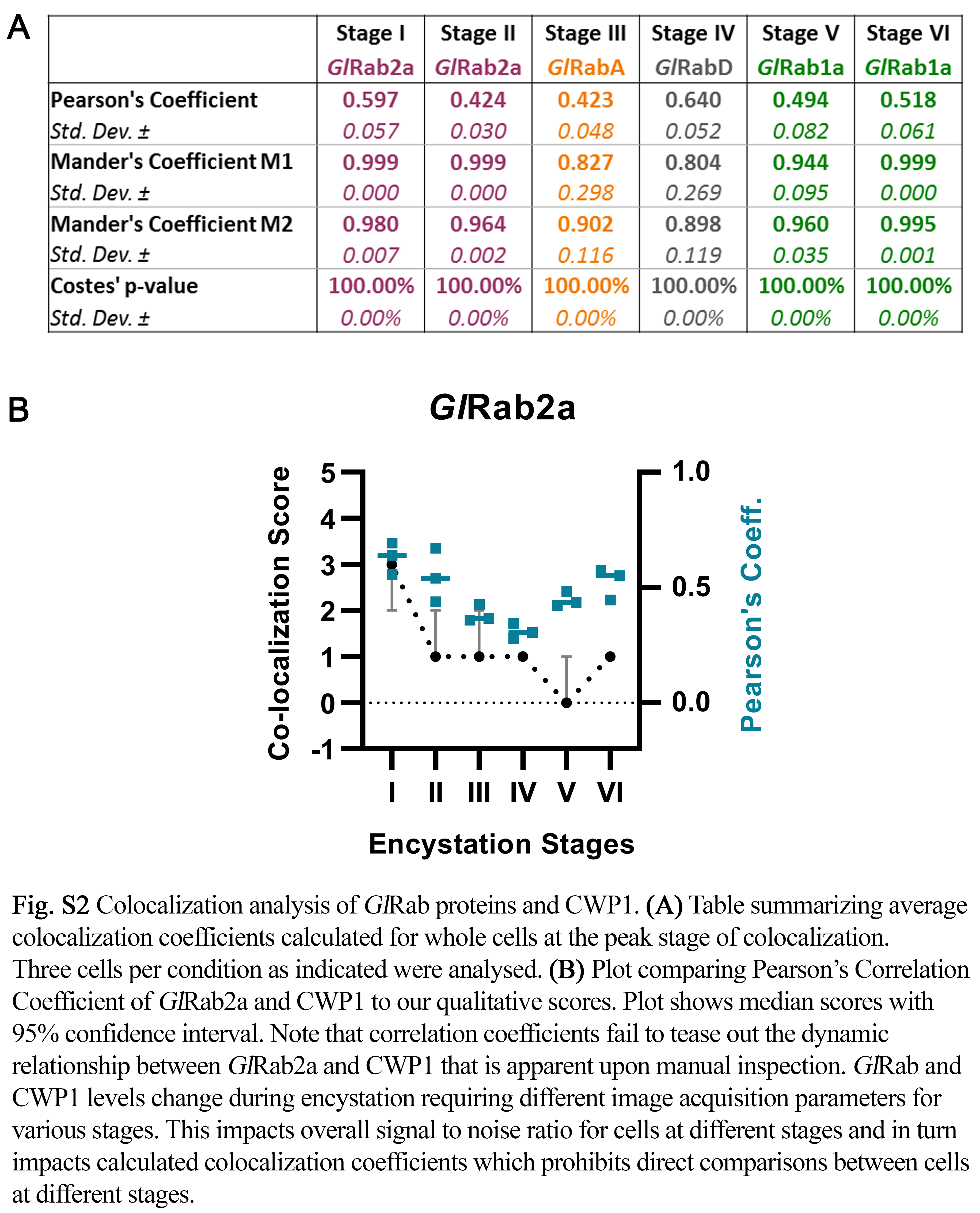

Supplement: Supplementary file 2 [file Image_2.tif]

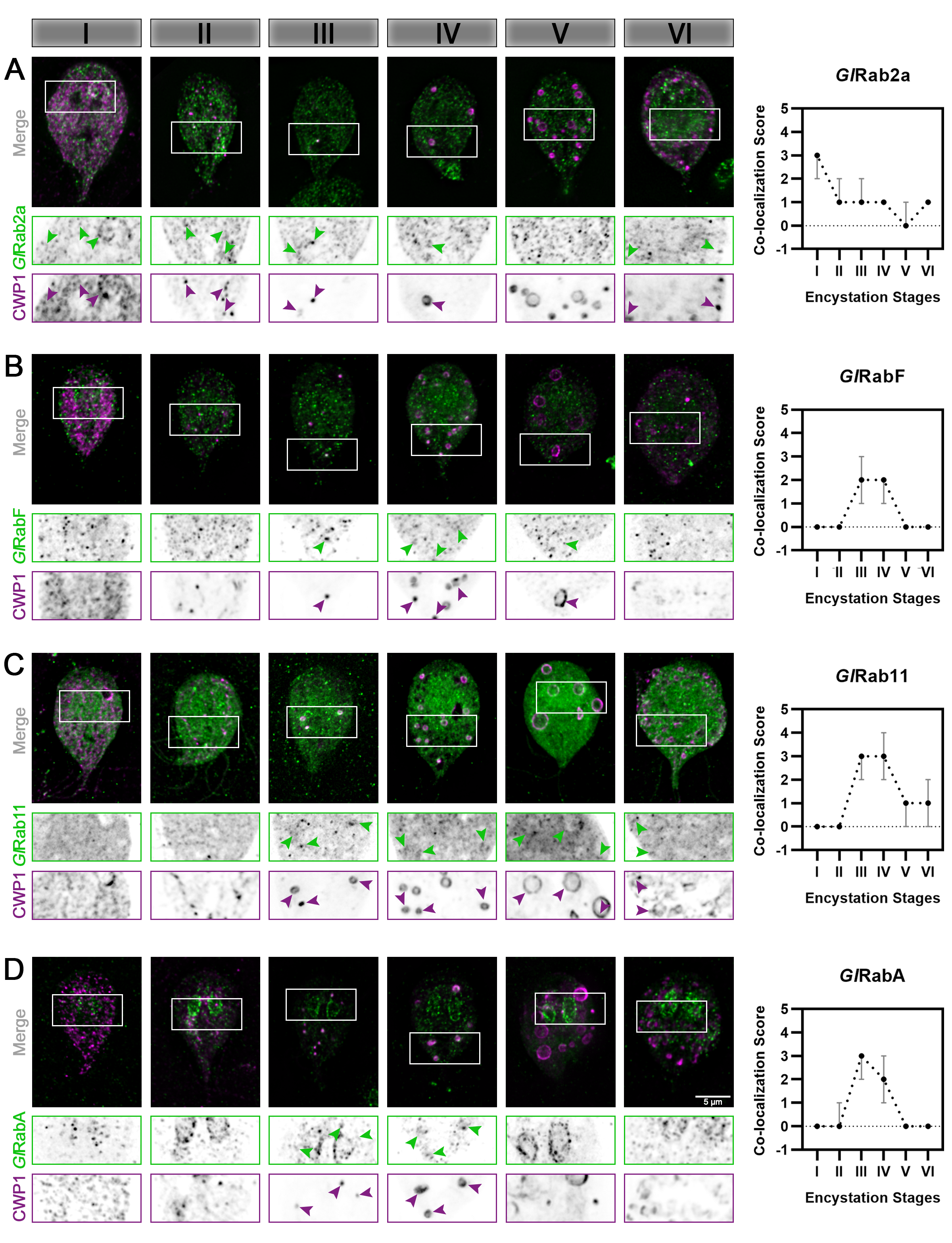

Supplement: Supplementary file 3 [file Image_3.tif]

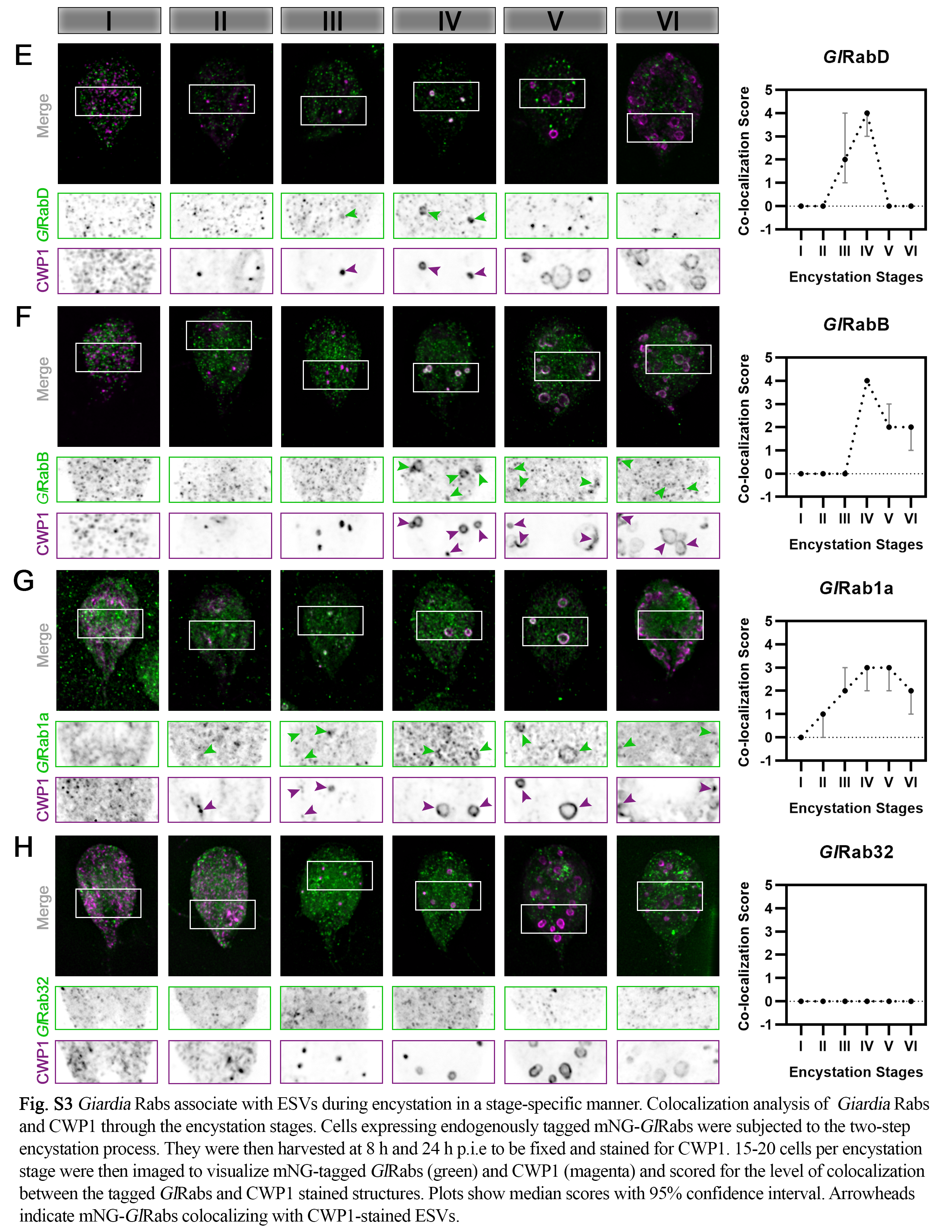

Supplement: Supplementary file 4 [file Image_4.tif]

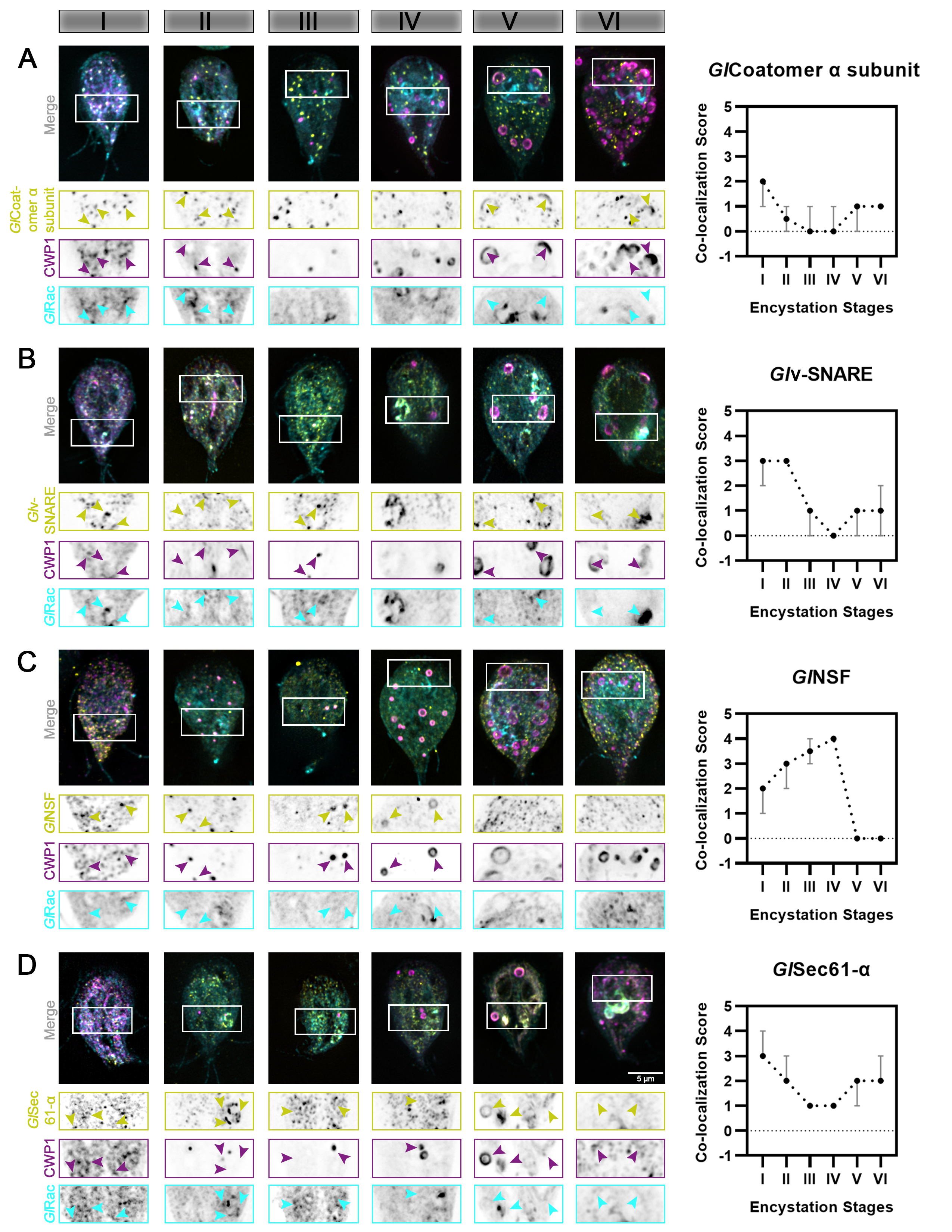

Supplement: Supplementary file 5 [file Image_5.tif]

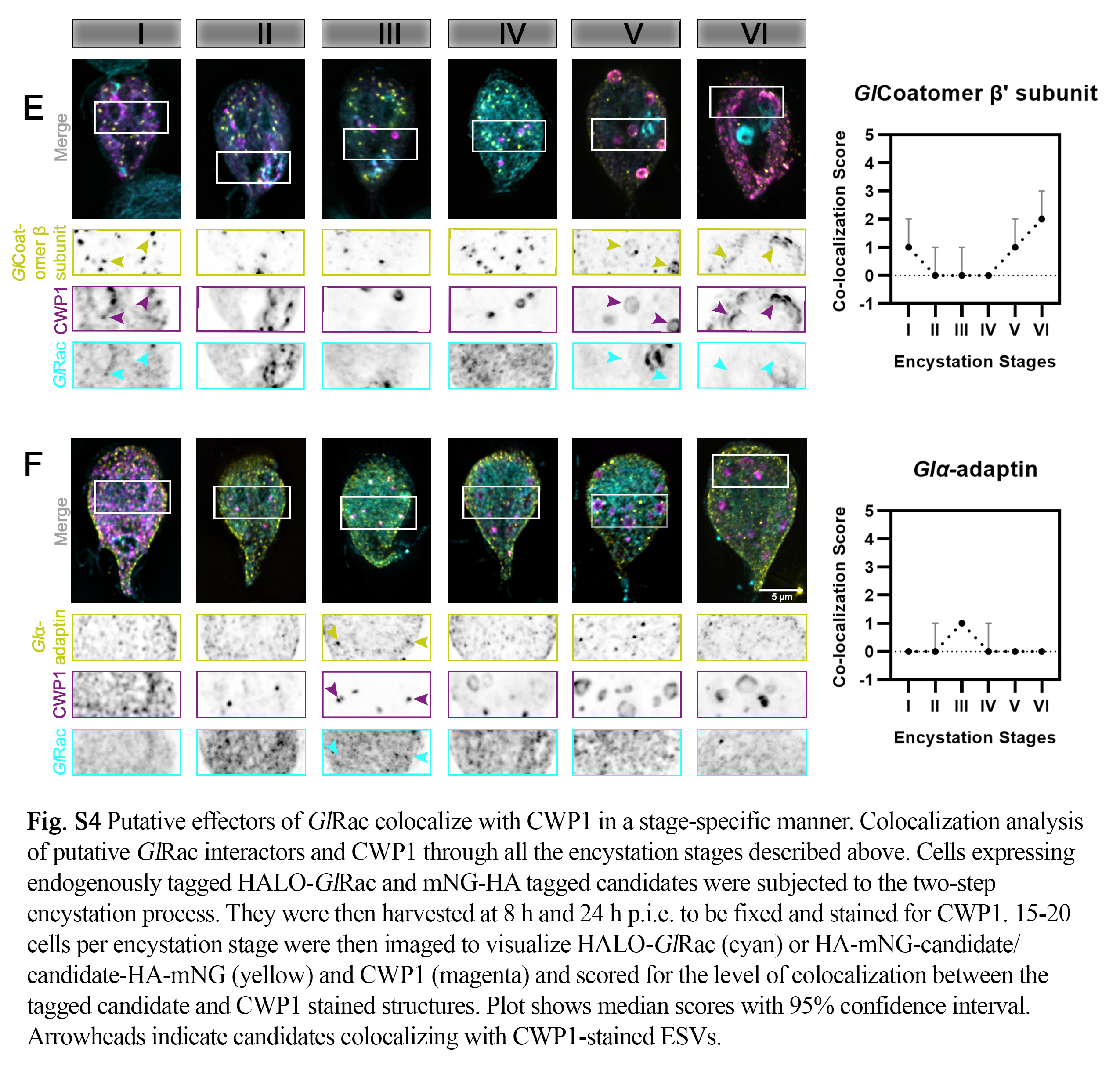

Supplement: Supplementary file 6 [file Image_6.tif]
